# The new era of academic hospitalist in Japan

**DOI:** 10.1002/jgf2.299

**Published:** 2020-01-30

**Authors:** Takashi Watari

**Affiliations:** ^1^ Postgraduate Clinical Training Center Shimane University Hospital Shimane Japan

## Abstract

Types of original research of peer‐reviewed publications by hospitalists.

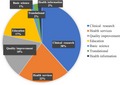

To the editor:

In Japan, the definition and position of general practitioners active in hospitals (hospitalists) are very vague, and their roles are not clearly organized in the new specialist system. Unfortunately, this situation is not specific to Japan; additionally, one of the largest group of doctors in the United States, hospitalists, also had this experience until recently.^1^, ^2^ A paper titled “Zero to 50 000 — The 20th anniversary of the hospitalist” was published in the New England Journal of Medicine in 2016. The paper suggests that hospitalists are already recognized as professionals in the United States, considering that there were 22 000 US cardiologists in the same year. Additionally, approximately 75% of tertiary academic hospitals in the United States adopted a system wherein a hospitalist was employed to treat inpatients.^1^ What enabled hospitalists to make a breakthrough in the United States? I believe that academic hospitalists substantially contributed to this breakthrough. Generally speaking, hospitalists work mainly in admission management and in wards with cross‐sectional perspectives, as though from a bird's‐eye view, as compared to the other specialists, who focus only on their fields. Specifically, the hospitalist works effectively in some important fields that extend across the hospital such as quality improvement, medical safety, clinical education after graduation, hospital management, and infection control, with the main focus being on common hospital admissions such as in general wards. Academic hospitalists, who are believed to account for over 15% of the hospitalists in the United States and work mainly in university hospitals, have actively contributed to educational and institutional research,^3^ and hospitalist teams are more effective than nonhospitalist teams in teaching residents and medical students in the ward.^3^, ^4^ Furthermore, as several studies indicate that it is more efficient for hospital management, more hospitals are beginning to adopt systems that excel in management, safety, and clinical education.^1^, ^2^, ^3^, ^4^ As a concrete example, to indicate the academic hospitalist's interest in clinical research as well as health services, a graph that has been modified from the original scientific paper by Do and colleagues is shown below (Figure 1).^5^ They presented research trends in hospital medicine with a systematic review of articles in PubMed. Their results revealed that special attention is paid to clinical research, health service research, medical quality and improvement, and medical education by the academic hospitalist. Hospitalist research does not necessarily have to include experimental medicine, nor does it have to be clinical research. Indeed, one advantage of hospitalists is that all events in the hospital can be studied. In Japan, relatively new working styles have emerged among hospitalists, and they are beginning to gain the trust of and good evaluations from specialists in other fields. We should encourage high‐quality outputs by generalists and improve their ability to communicate using the “same language” as professionals in other fields. It is also essential to develop Japanese academic hospitalists who can contribute to clinical and health service research and medical quality improvements.

**Figure 1:**
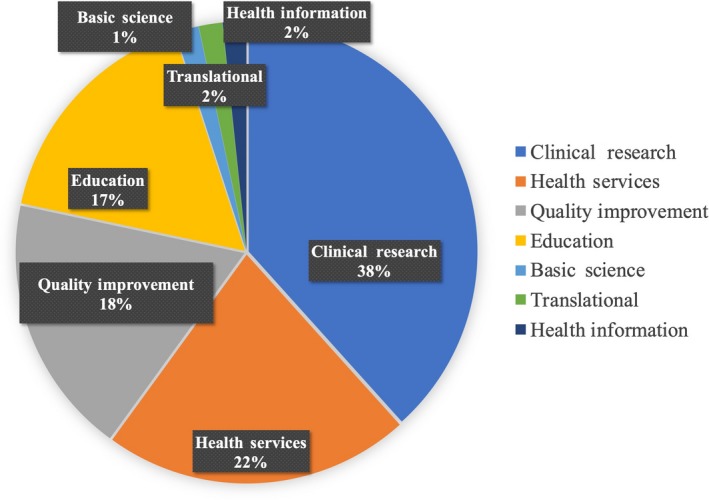
Types of original research of peer‐reviewed publications by hospitalists in 2013 (Modified from Reference^5^)

## CONFLICT OF INTEREST

The authors have stated explicitly that there are no conflicts of interest in connection with this article.

## FUNDING INFORMATION

Author TW was supported by grants by JSPS KAKENHI Grant Number 17K15745.
